# A Faculty-Constructed AI Tutor for Personalized Learning and Remediation in a U.S. PharmD Immunology Course: An “In-House” Evaluation of New Learning Technology

**DOI:** 10.3390/pharmacy14020059

**Published:** 2026-04-03

**Authors:** Ashim Malhotra

**Affiliations:** Department of Pharmaceutical and Biomedical Sciences, California Northstate University College of Pharmacy, 9700 W Taron Drive, Elk Grove, CA 95758, USA; ashim.malhotra@cnsu.edu; Tel.: +1-(916)-686-8885

**Keywords:** AI tutor, AI-enabled learning, AI-based remediation, AI-based forecasting and predictive analytics

## Abstract

While generative AI becomes increasingly available in higher education, faculties find it challenging to design, implement, and evaluate AI-enabled personalized learning systems within accreditation-constrained professional curricula. This method paper describes ADAPT (Assessment-Driven AI for Personalized Tutoring), a home-grown AI tutoring and remediation ecosystem implemented in a required PharmD immunology course. Using standard learning management (Canvas) and assessment (ExamSoft) platforms, a 20-item quiz mapped to six immunology mastery domains (N = 34; mean 69.1%, SD 17.9; Cronbach’s α = 0.73) was used to trigger tiered, structured generative AI remediation at both individual student and cohort levels. Instructional impact was evaluated using reliability indices, item-level difficulty analyses, and paired pre/post-assessment comparisons. Following AI-guided remediation, mean performance increased to 79.8% (+10.7 percentage points), variability decreased (SD 14.4), and assessment reliability improved (ExamSoft KR-20 0.87) compared with the diagnostic exam, the first midterm exam, and the final exam, respectively. Item difficulty stabilized (mean ≈ 0.80), with sustained retention of targeted concepts on the final examination. ADAPT provides a replicable, low-cost methodological blueprint for faculties to independently construct assessment-driven AI tutoring systems and lays the foundational steps for future AI-based predictive analysis workflow for at-risk students.

## 1. Introduction

The field of higher education, especially health professions education, stands to gain tremendously from generative AI and Intelligent Systems that have the potential to deliver context-aware, adaptive, targeted, and personalized learning environments [1]. While recent investigations by Demartini et al. and Gligorea et al. provide important evidence regarding the efficiency of generative machine learning in enhancing student learning and comprehension by almost 25% [2,3], the three most common modalities of AI-based education tools for (1) customized learning pathways, (2) AI-integrated competency-based learning, and (3) personalized feedback rely on expensive, large-scale, and engineering-based model construction, producing the prevailing AI tools on “the shelf” [1]. However, the AI- and machine-language-based educational literature lacks documentation of an instructor-led, low-cost, context-aware, adaptive, and personalized learning system that directly results in student learning gains. While commercially developed off-the-shelf AI solutions may emerge in some professions, offering a higher-cost/higher-quality product, due to relatively modest student numbers globally and regional variations in practice, pharmacy programs are unlikely to be a major target for such solutions. Even if such solutions become available, financial constraints in many programs necessitate the pursuit of low-cost in-house solutions where possible.

In the U.S., ACPE Standards 2025, Appendix 1, and COEPA emphasize the acquisition of learner-centered technical knowledge and skills in all pharmacy programs. This requires seamless intellectual integration between foundational sciences and clinical decision-making, with the former continuously informing the latter [4,5]. However, PharmD students face significant challenges with (1) spaced memory retrieval [6], (2) self-directed learning [7], (3) concept mastery [8], (4) integration [9], and (5) the meaningful application of foundational data science for real-life clinical scenarios, especially for courses such as immunology [10]. ADAPT was constructed to allow students to self-assess their weaknesses in concept retrieval and integration with foundational science and immunology concepts, with clinical practice through assessment-anchored AI-led personalized tutoring, which would engage students, premised on Astin’s theory of student engagement [11].

In this method paper, we report on the design, deployment, and assessment of ADAPT, an assessment-driven AI tutoring framework. ADAPT is a faculty-constructed, adaptable, cost-effective, personalized, and targeted generative-AI-based learning ecosystem. ADAPT’s overarching goal was to enhance PharmD students’ engagement, memory retrieval, concept mastery, and foundational and clinical science integration. A secondary goal was to create a learner-centered, self-directed, metacognitive AI tool that provided real-time data for students to self-assess, followed by adaptive and targeted interventions to help students relearn missed concepts.

To achieve this, we incrementally constructed ADAPT as a scalable large language model (LLM)-based multimodal platform with three integrated components through an instructor-orchestrated, quiz-triggered LLM tutoring integrated into standard PharmD course infrastructure (Canvas and ExamSoft), with auditable artifacts and downstream exam performance signals. This is markedly different from the AI-based personalized learning field, which is largely moving towards algorithmic personalization through recommender systems (content/video/resource recommendations), learning analytics, and adaptive pathway optimization, progressing over time toward natural language processing and, most recently, LLM-enabled content generation (e.g., ChatGPT-era “autonomous content generation” and “smart tutoring”). Our work shows that ADAPT led to a 10% early gain and the identification of missed domains by students. The step-by-step design pathway, including the Python code for construction and an assessment matrix, is described.

## 2. Methods

### 2.1. Study Context and Course Setting

This study was conducted in the fall of 2025 in a required immunology course within the ACPE-accredited four-year Doctor of Pharmacy (PharmD) program at California Northstate University College of Pharmacy. The 3-credit-hour course enrolls a diverse cohort of professional students (N = 39), including a substantial proportion of first-generation, multilingual, and international students. Instruction is delivered using a combination of didactic lectures, active-learning exercises, and technology-supported formative assessments delivered through the Canvas learning management system and ExamSoft assessment platform.

The P3 Immunology and Rheumatology PBS803 course is arranged in three sequential modules: (1) Introduction to Immunology, (2) Immune Diseases, Hypersensitivity, and Autoimmune Diseases, and (3) Immunopharmacology, including immunomodulators and immunosuppressive agents for solid organ transplantation. The course includes topics mapped to Appendix 1 of the ACPE Standards 2025, 100% coverage of the relevant Tier 1 topics of the 2025 AACP Tool Kit, and about 80% coverage of Tier 2 topics. This foundational science course is followed by a pharmacotherapeutics course in the spring semester of the third professional year, where dosing and PPCP become the primary immune focus.

The college utilizes a host of active-learning strategies, including Team-Based Learning pedagogy. Each 3 h long class commences with an individual readiness assurance test (iRAT), followed by a team readiness assurance test (tRAT), a lecture emphasizing core concepts, and an application exercise. Students are formally evaluated in Midterm 1, Midterm 2, and a final examination.

The AI tutor intervention and evaluation focused on the first immunology module of the course, which emphasizes foundational immune mechanisms, including immune organs, central immunology concepts, T- and B-cell development and activation, antigen capture and presentation, immune signaling pathways, and cytokine-mediated responses.

### 2.2. Instructional Design Framework

The AI tutor ecosystem was designed as a “diagnostic quiz”-informed, adaptive instructional system grounded in principles of targeted remediation and cognitive flexibility [12]. At our institution, the immunology course is offered to third-year professional students in a four-year, accredited Doctor of Pharmacy (PharmD) program. The course is organized in three modules and moves from introductory concepts, anatomy/physiology, and cellular basis (Module 1) to pathology and immune disorders (Module 2) and pharmacology of drugs and pharmacotherapeutics (Module 3). Each module (except Module 3) ends with a “midterm exam.” The premise was for students to take a diagnostic self-assessment a week before the first midterm examination, identify the missed concepts through incorrectly answered questions based on 70%-threshold mastery, and use AI tutoring that would provide a customized pattern of repeated tutoring, different for each student. Conceptually, the design integrates elements of Astin’s student engagement theory [11] and cognitive flexibility theory [12], emphasizing iterative exposure, multiple representations of complex concepts, and learner-directed remediation based on demonstrated need rather than uniform review.

**Figure 1:**
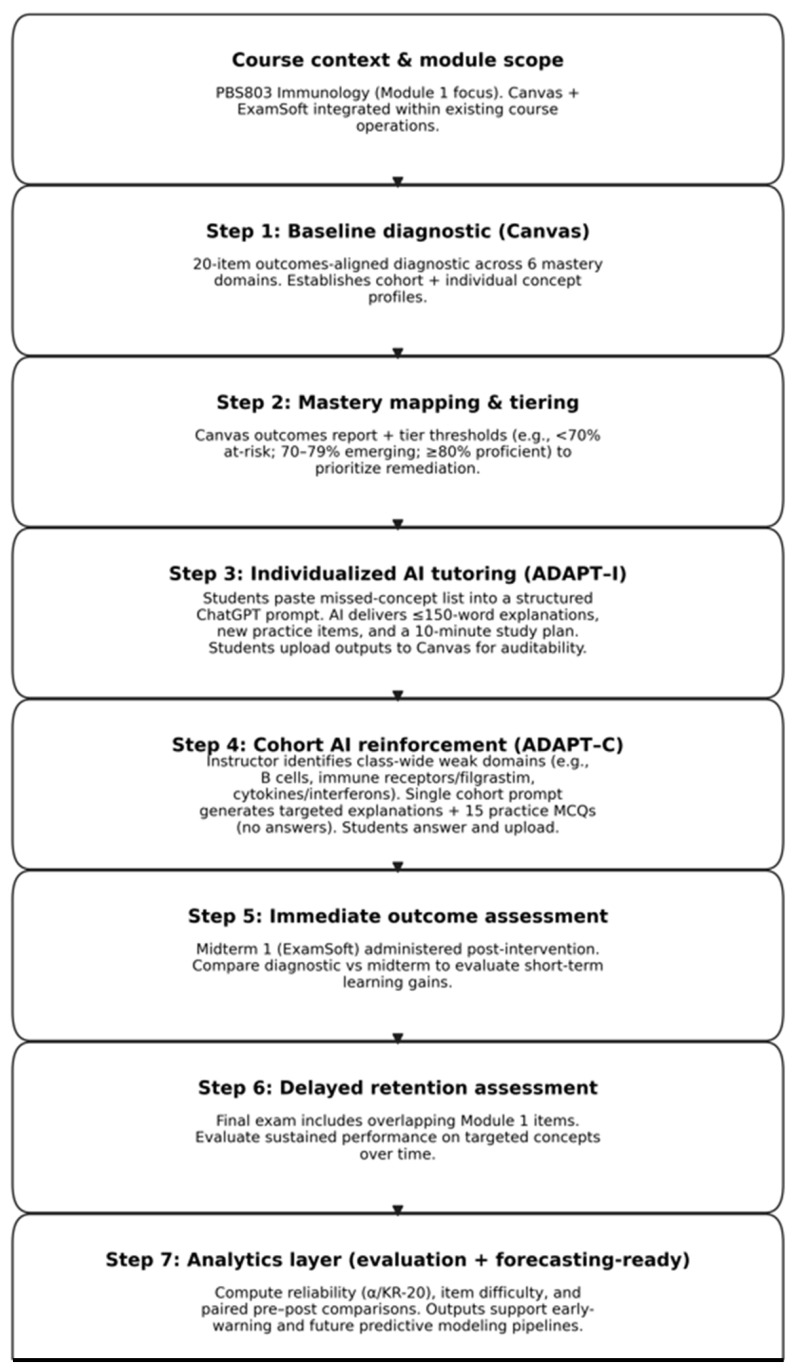
ADAPT program workflow. The figure depicts how ADAPT progresses from diagnostic insight to tiered mastery mapping, individualized and cohort-based remediation, outcomes evaluation, and forecasting-ready analytics.

Rather than functioning as a stand-alone instructional tool, the AI tutor was embedded within the existing course infrastructure and used to augment instructor-led teaching by responding dynamically to diagnostic performance signals at both the individual and cohort levels. Figure 1 (workflow schematic) summarizes the stepwise instructional design and assessment flow.

### 2.3. Diagnostic Assessment (Quiz) and Mastery Mapping

A 20-item diagnostic quiz was administered via Canvas one week before Midterm 1 to assess baseline understanding across six predefined immunology mastery domains aligned with course learning outcomes. Thus, the AI intervention was placed after completion of 6 lectures, during which students had practiced formative quizzes (iRATs/tRATs) following pre-reading assignments and classroom lectures. The six concept mastery categories directly related to the 6 lectures and included (1) antibody structure, classes, and therapeutics, (2) antigen capture and presentation and vaccines, (3) B-cell development and activation, (4) immune cells and receptors and drugs such as filgrastim, (5) inflammation, cytokines, and IFN-based drugs in multiple sclerosis, and (6) innate and adaptive immunity and immune organs. Diagnostic items were mapped to specific conceptual categories (e.g., B-cell development, antibody–antigen interactions, immune signaling), allowing both student-level and cohort-level mastery profiling. We also introduced challenging topics such as student-led discussions on immunological responses to orphan diseases like Barth Syndrome, based on mitochondrial disease mechanisms [13], to encourage the cohort to think about acute and chronic changes to the inflammatory milieu.

Diagnostic performance data were analyzed to identify (1) individual students with concept-specific deficiencies and (2) content domains exhibiting widespread underperformance across the cohort. These diagnostic outputs served as the trigger for subsequent AI-guided remediation.

#### Performance Tiering and Diagnostic Thresholds

Diagnostic quiz performance was further operationalized using a tiered mastery framework to support structured remediation and downstream performance interpretation. Based on Canvas-generated outcome analytics, students were categorized into three performance tiers using predefined thresholds: Tier 1 (≥80% mastery), Tier 2 (70–79% mastery), and Tier 3 (<70% mastery). The 70% threshold was selected a priori as the minimum acceptable level of conceptual mastery aligned with course expectations and accreditation benchmarks. Tier assignments were used to (i) prioritize individual-level AI tutoring for students in Tier 3, (ii) identify concepts requiring cohort-level reinforcement when a substantial proportion of students clustered below the threshold, and (iii) examine shifts in score distributions and lower-tail compression following AI-guided remediation. Tiering was applied as an instructional decision rule rather than as a summative evaluative label.

### 2.4. AI-Guided Remediation Interventions

#### 2.4.1. Individual-Level AI Tutoring

Following the diagnostic assessment, students were instructed to engage with a structured AI tutoring activity using guided prompts provided by the instructor. Prompts required students to (1) explicitly identify to ChatGPT 5.0 the concepts they had missed, (2) request targeted explanations, and (3) generate new practice questions with feedback. Students submitted AI-generated explanations and responses through Canvas, creating an auditable record of the AI tutoring, which was examined by the course instructor.

Importantly, students were encouraged to interact with the AI tutor in a language of their choice, enabling multilingual support for learners with diverse linguistic backgrounds. Although usage frequency by language was not formally quantified, this design feature was intentionally incorporated to reduce language-related cognitive load and improve accessibility.

#### 2.4.2. Cohort-Level AI Reinforcement

In addition to individualized remediation, a second AI-guided intervention was implemented based on cohort-level diagnostic analysis following student upload of AI tutoring materials. Domains with consistently low diagnostic performance for the entire class were addressed through a class-wide AI prompt instructing the system to reteach these concepts and generate additional practice questions. Students also had to upload the new AI-targeted tutoring into Canvas. This second AI intervention was designed to reinforce shared conceptual gaps prior to Midterm 1 and to model structured AI-supported learning strategies for students.

### 2.5. AI Prompt Architecture and Layered Tutoring Logic

The AI tutor ecosystem was implemented as a two-layer, prompt-driven instructional system designed to translate diagnostic assessment signals into targeted, adaptive learning interventions without custom software development. Rather than relying on opaque, model-driven personalization, the system operationalized instructor-defined logic through structured prompts that functioned as modular control layers.

At the individual level (Layer 1), diagnostic outputs were mapped to student-specific knowledge gaps. Each student started an AI tutoring session, in our case, in ChatGPT 5.0 (OpenAI, San Francisco), by passing their missed questions as inputs into a standardized prompt template that constrained scope, depth, and output format. This prompt enforced (i) concise conceptual explanation, (ii) deliberate practice generation, and (iii) time-bound study planning. The logic of this layer can be represented as follows:
------------------------------------------------ def individual_ai_tutor(missed_questions):  system_role = “Immunology AI Coach for a third-year PharmD student”  constraints = {    “max_explanation_length”: 150, # words per concept    “practice_questions_per_item”: 3,    “include_answers”: True,    “study_plan_duration”: “10 min”  }   prompt = f”““  You are my Immunology AI Coach.  Explain each of the following missed questions and their correct answers in ≤{constraints[‘max_explanation_length’]} words.  For each question, generate {constraints[‘practice_questions_per_item’]} new practice questions (with answers).  Then suggest a {constraints[‘study_plan_duration’]} focused review plan.   Missed Questions:  {missed_questions}  “““  return prompt --------------------------------------------------

This design ensured student-directed remediation while preserving instructor control over cognitive load, rigor, and assessment alignment. All AI outputs were submitted through Canvas, creating an auditable artifact of AI-student interaction.

At the cohort level (Layer 2), aggregated diagnostic performance identified content domains with widespread underperformance. A second prompt layer was deployed to reinforce these concepts uniformly across the class, independent of individual errors. This prompt emphasized conceptual synthesis, clinical reasoning, and exam-relevant practice, and intentionally withheld correct answers to promote active retrieval:
--------------------------------------------------- def cohort_ai_reinforcement(target_topics):  constraints = {    “topics”: target_topics,    “max_explanation_length”: 150,    “questions_per_topic”: 5,    “include_answers”: False,    “student_level”: “Third-year PharmD”,    “study_plan_duration”: “10 min”  }   prompt = f”““  Explain the following topics, each in ≤{constraints[‘max_explanation_length’]} words:   {constraints[‘topics’]}   Then create {constraints[‘questions_per_topic’]} multiple-choice questions per topic  (four options each, no correct answers provided).  Questions should assess mechanisms, clinical application, and immunological reasoning.   Finally, generate a {constraints[‘study_plan_duration’]} study plan to prepare for a midterm exam.  “““  return prompt -------------------------------------------------

This layered architecture allowed the same AI model to function in two distinct instructional modes, (1) precision remediation (individual layer designed for targeted tutoring) and (2) structured reinforcement (cohort layer), both triggered by assessment-derived signals rather than continuous AI surveillance.

Students were permitted to interact with the AI in a language of their choice, enabling multilingual explanations and practice generation. While language-specific usage metrics were not formally captured, this feature was intentionally embedded to reduce linguistic cognitive load in a diverse, multilingual PharmD cohort.

Critically, the AI tutor did not replace instructor decision-making; instead, it served as a scalable execution layer that operationalized instructor-defined pedagogical intent. The system is therefore replicable across courses and institutions using standard learning management systems and publicly available large language models, without reliance on proprietary platforms or engineering support.

### 2.6. Outcome Assessments

Three assessment points were analyzed: (1) Diagnostic Quiz (Canvas): Baseline assessment of mastery prior to AI intervention; (2) Midterm 1 (ExamSoft): High-stakes assessment administered following AI-guided remediation, measuring immediate post-intervention performance; and (3) Final Examination (ExamSoft): Cumulative assessment including overlapping immunology module items, used to evaluate delayed retention of targeted content. The midterm and final examinations were constructed independently of the diagnostic quiz but aligned with the same mastery domains to permit longitudinal comparison of conceptual performance. The present study establishes the diagnostic and instructional backbone required for predictive analytics; formal model-based prediction is proposed as a subsequent phase.

### 2.7. Statistical Analysis

Descriptive and inferential analyses were conducted to examine performance changes across assessment points. Internal consistency reliability was estimated using Cronbach’s alpha for the diagnostic quiz and KR-20 for exam-based assessments. Item-level difficulty indices were calculated for all assessments to evaluate changes in content mastery and score distribution characteristics. Paired comparisons of diagnostic and Midterm 1 performance were conducted at the cohort level to assess the magnitude and direction of change. Score distributions, variability, and shifts in lower-tail performance were examined using box-and-whisker plots and frequency-based change analyses. Retention was evaluated by comparing item difficulty and performance patterns for overlapping immunology content between Midterm 1 and the final examination. All analyses were conducted using two-tailed statistical tests with an alpha threshold of 0.05. Given the instructional design and absence of randomization, analyses were intended to support feasibility, instructional impact, and longitudinal performance characterization rather than causal inference.

Overall, this methodological approach emphasizes replicability, instructor agency, and minimal resource burden, demonstrating how AI-enabled adaptive learning systems can be constructed and evaluated within standard curricular infrastructures without specialized software development or external engineering support.

## 3. Results

### 3.1. Diagnostic Identification of Individual- and Cohort-Level Learning Gaps

**Figure 2:**
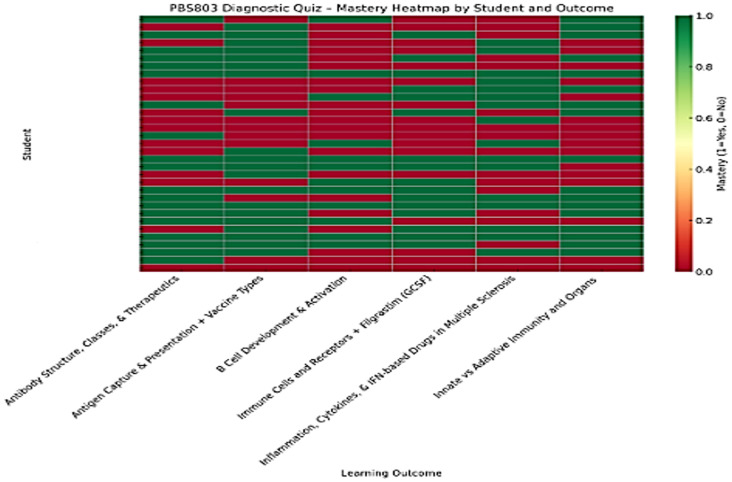
Student-level diagnostic mastery heat map (baseline assessment). Figure 2 presents a student-level heat map summarizing performance on the 20-item diagnostic quiz administered prior to AI intervention. Each row represents an individual student (identifiers removed), and each column represents one of six immunology mastery domains.

Following the administration of the 20-item diagnostic quiz (N = 34), baseline performance demonstrated substantial variability (mean 69.1%, SD 17.9), with acceptable internal reliability (Cronbach’s α = 0.73). Figure 2 presents an anonymized heat map of individual student performance across core immunology domains. Visual inspection reveals concentrated areas of low mastery shared across many students, rather than isolated individual deficits. In particular, repeated low-performance clusters were observed in domains related to B-cell development and activation, antigen capture and antibody biology, and immune signaling pathways.

**Figure 3:**
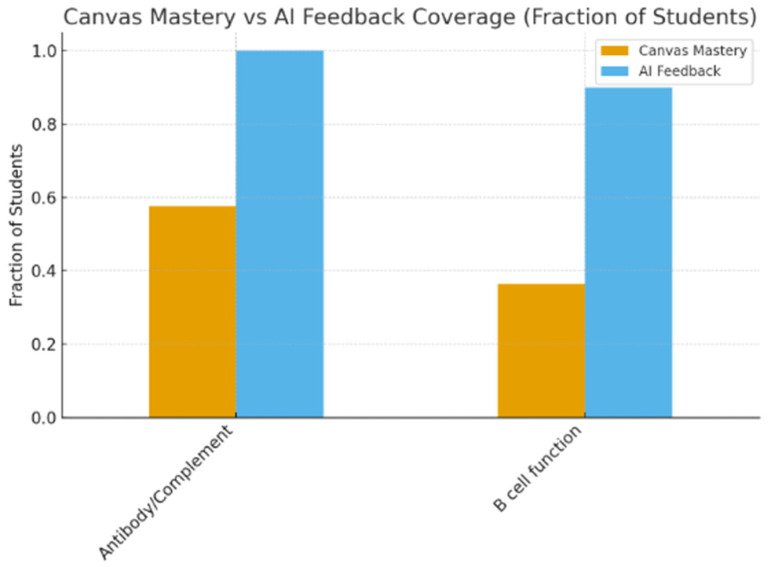
Cohort-wide diagnostic gaps and AI remediation focus areas. Figure 3 aggregates diagnostic performance across the cohort to identify immunology domains with the highest frequency of incorrect responses. Bars representing diagnostic underperformance (warm colors) are contrasted with domains that subsequently triggered the greatest volume of AI-guided tutoring activity (cool colors). B-cell development and activation, immune cells and receptors (including filgrastim/G-CSF), and inflammation/cytokine signaling emerged as the primary cohort-level targets for AI reinforcement.

To further characterize these deficiencies at the concept level, Figure 3 aggregates diagnostic item performance across the cohort. Two concept clusters, B-cell biology and antigen–antibody interactions, emerged as the most frequently missed domains, with multiple items falling below 60% correct. These same domains also corresponded to the highest volume of AI-generated tutoring prompts, indicating alignment between diagnostic signal and AI-guided instructional targeting.

**Figure 4:**
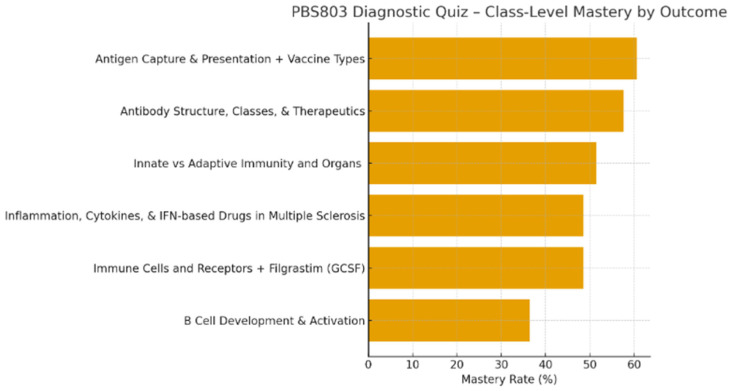
Cohort-level mastery distribution across immunology domains. Figure 4 summarizes cohort-level mastery by immunology domain following the diagnostic assessment. This visualization shifts the analytic focus from individual learners to shared conceptual vulnerabilities, enabling implementation of a second, cohort-level AI tutoring intervention designed to reinforce commonly missed concepts prior to Midterm 1.

Figure 4 summarizes cohort-level diagnostic performance across all major immunology categories. Consistent with the individual-level heat map, these results confirm that observed weaknesses were systemic at the cohort level, rather than driven by a small subset of students.

Based on these findings, a second AI-guided intervention was implemented at the cohort level, in which students prompted the AI to reteach the lowest-performing concepts and generate additional practice questions. This two-tiered approach, consisting of individualized remediation followed by cohort-wide reinforcement, formed the instructional backbone of the intervention phase.

### 3.2. Performance Change Following AI-Guided Intervention

Immediate post-intervention performance was assessed using Midterm 1 (N = 33). Mean performance increased to 79.8%, representing a +10.7 percentage-point gain relative to the diagnostic baseline, with reduced variability (SD 14.4). Assessment reliability improved substantially (KR-20 = 0.87), suggesting increased measurement stability following instruction.

**Figure 5:**
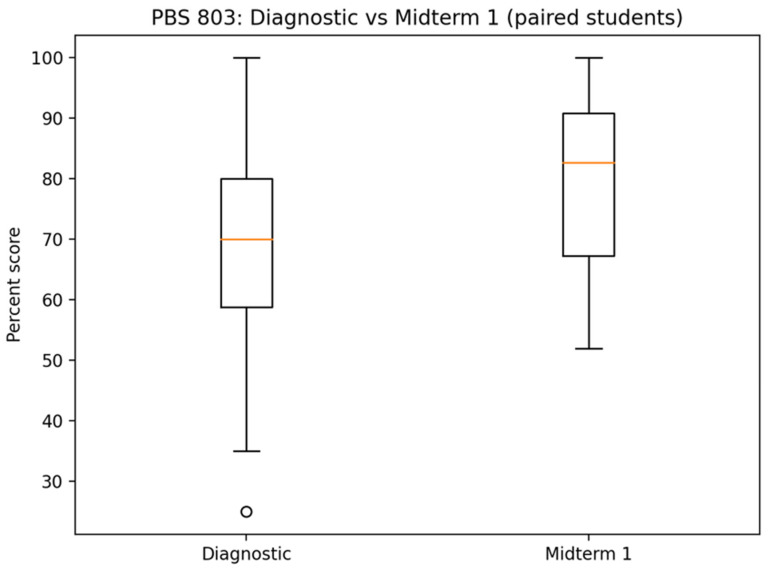
Pre- and post-intervention performance comparison (diagnostic vs. Midterm 1). Figure 5 displays box-and-whisker plots comparing overall student performance on the diagnostic quiz and Midterm 1. The orange line depicts the median. Following individualized and cohort-level AI tutoring, median scores increased and score dispersion narrowed, indicating both improvement in central tendency and compression of lower-tail performance after the intervention.

Figure 5 displays paired student performance on the diagnostic quiz and Midterm 1. The distribution demonstrates a clear upward shift in median performance, accompanied by compression of the lower tail. While a small number of students showed minimal or negative change, the majority exhibited moderate-to-large gains.

**Figure 6:**
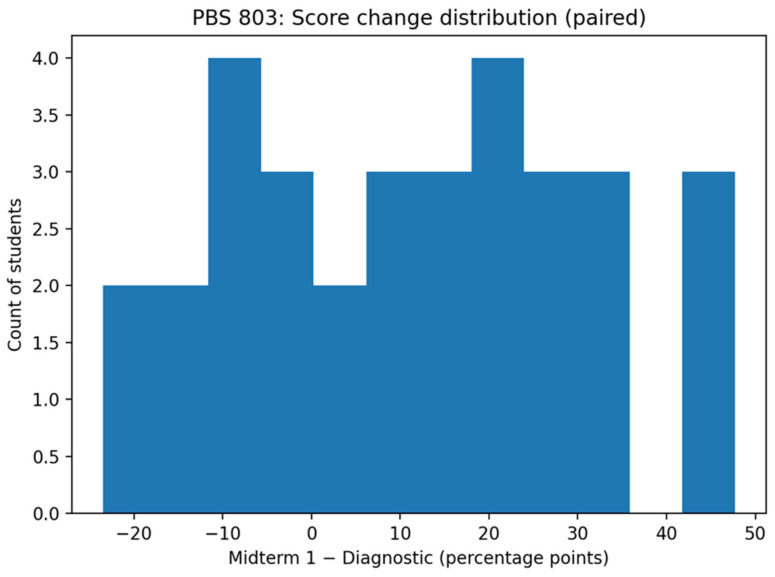
Distribution of individual score changes following AI tutoring. Figure 6 illustrates the distribution of individual student score changes between the diagnostic quiz and Midterm 1. Positive values indicate performance gains, while negative values indicate score decreases. The distribution demonstrates that the majority of students experienced meaningful score improvements following AI-guided remediation, with relatively few students showing substantial declines.

Figure 6 illustrates the distribution of individual score changes (Midterm 1 minus diagnostic). Most students demonstrated positive gains, with several improvements exceeding 20 percentage points. Negative changes were limited in number and magnitude, indicating that performance gains were not driven solely by a small subset of high improvers.

### 3.3. Item-Level Stabilization and Retention of Targeted Concepts

**Figure 7:**
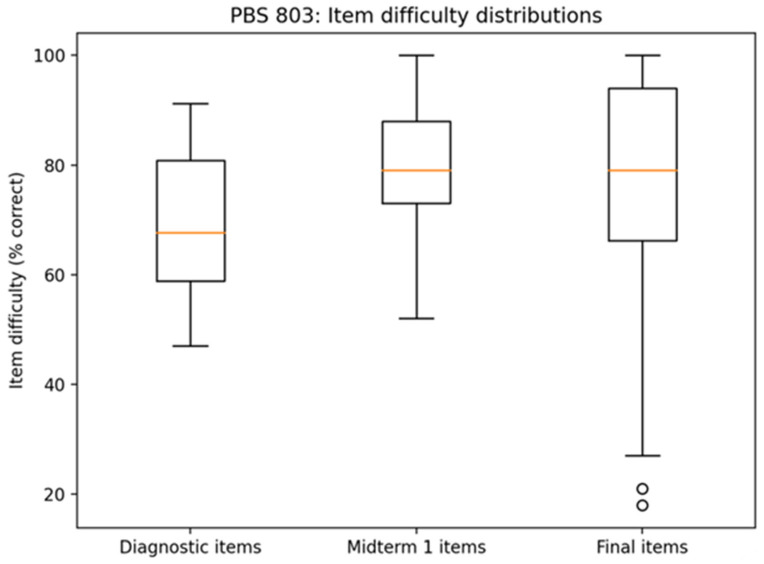
Item difficulty and retention of targeted concepts on the final examination. Figure 7 compares item difficulty indices for overlapping immunology concepts assessed on Midterm 1 and the final examination. The orange line depicts the median value. Concepts targeted through AI-guided remediation maintained moderate-to-high item difficulty values on the final exam, suggesting durable retention of previously weak domains rather than short-term performance inflation.

Item difficulty analyses further contextualized these performance shifts. As shown in Figure 7, diagnostic items exhibited wide variability in difficulty, with several items below 60% correct. In contrast, Midterm 1 items demonstrated a higher and more compressed difficulty distribution (mean item difficulty approximately 0.80), with only 2 of 52 items falling below 60% correct.

Importantly, analysis of overlapping immunology items on the final exam indicated sustained performance on Module 1 concepts. Item difficulty for retained concepts remained in the mid-to-high 70% range or above, with no re-emergence of the low-performing clusters observed at baseline. This pattern suggests durable conceptual mastery rather than short-term test preparation effects.

### 3.4. Summary of Observed Performance Trajectory

Across analyses, a consistent performance trajectory was observed: (1) diagnostic identification of concentrated conceptual weaknesses; (2) targeted AI-guided remediation aligned to those weaknesses; (3) improved mean performance and reduced variability on Midterm 1; and (4) maintenance of item-level performance on the final exam. While causal attribution at the individual level cannot be inferred, these findings support the feasibility and instructional value of a low-cost, AI-enabled diagnostic-remediation system and establish an empirical foundation for future work integrating predictive analytics into PharmD education.

## 4. Discussion

### 4.1. Purpose and Framing of the Present Work

The primary contribution of this study is methodological rather than experimental. The intent was not to establish causal efficacy of artificial-intelligence-based tutoring, nor to evaluate student outcomes as a human subject investigation, but rather to document and empirically demonstrate a replicable instructional architecture, ADAPT, that enables assessment-driven personalization using existing educational infrastructure. Student performance data are presented solely to illustrate functional operation, feasibility, and instructional alignment of the system, consistent with methods-focused scholarship in health professions education.

Accordingly, the results should be interpreted as “proof-of-function” rather than “proof-of-effect,” providing evidence that the proposed workflow can be implemented by faculty at low to no cost, generate actionable diagnostic signals, and translate those signals into targeted learning interventions without specialized software development.

### 4.2. Empirical Signals Supporting Functional Validity of the ADAPT Framework

Although not designed as an outcomes trial, the observed performance patterns provide important confirmation that the ADAPT workflow operates as intended. Baseline diagnostic performance revealed substantial heterogeneity in foundational immunology mastery (mean 69.1%, SD 17.9; reliability α = 0.73), with multiple items falling below 60% correctness and clear clustering of deficiencies in B-cell development, antigen capture, and immune signaling domains. These diagnostic signals directly informed both individualized and cohort-level AI remediation.

Following implementation of the ADAPT-guided interventions, Midterm 1 performance demonstrated a 10.7 percentage-point increase in cohort mean performance (79.8%), accompanied by reduced score variability (SD 14.4) and improved internal consistency (KR-20 = 0.87). At the item level, mean difficulty indices increased to approximately 0.80, with only 2 of 52 items remaining below the 60% threshold. Importantly, domains that exhibited widespread diagnostic weakness did not demonstrate downstream collapse, indicating stabilization of previously fragile concepts.

Delayed performance patterns further support the functional coherence of the system. Overlapping immunology content on the final examination maintained mid-to-high item difficulty levels, with no re-emergence of low-performing clusters identified in the diagnostic phase. While not constituting a formal retention study, these findings suggest that the ADAPT workflow did not merely facilitate short-term score inflation but supported durable conceptual reinforcement, consistent with prior work on targeted remediation and iterative exposure in complex biomedical domains.

### 4.3. Relationship to Prior AI-Enabled Personalized Learning Approaches

Existing empirical studies of AI-enabled personalized learning in medical and health professions education have largely focused on platform-driven intelligent tutoring systems [1], adaptive learning environments [14], or generative AI “teaching assistants” developed as stand-alone tools [15]. These systems often rely on continuous learner surveillance, behavioral analytics, or proprietary architectures to deliver personalization [16,17,18,19].

In contrast, ADAPT advances the field by demonstrating a faculty-defined, assessment-triggered personalization model that operates independently of algorithmic black boxes. Personalization in ADAPT is explicitly rule-based: diagnostic performance is mapped to predefined mastery domains, tiered thresholds guide instructional response, and AI interactions are constrained through instructor-authored prompts aligned with assessment expectations.

This distinction is non-trivial. While prior work has shown that AI tutors can improve learning outcomes, few studies provide transparent, instructor-controllable methods for translating assessment data into personalized learning actions that can be readily replicated across institutions. By externalizing the logic of personalization rather than embedding it within opaque models, ADAPT prioritizes instructional agency, auditability, and scalability, which remain underdeveloped in much of the existing literature.

### 4.4. Instructional Theory and Pedagogical Alignment

The ADAPT framework is theoretically anchored in Astin’s student engagement theory [11] and Spiro’s cognitive flexibility theory [12], both of which emphasize active learner participation, adaptive exposure, and iterative restructuring of knowledge. The requirement that students explicitly identify missed concepts, generate new practice questions, and submit AI-mediated outputs transforms AI use into an engaged, metacognitive activity, rather than passive content consumption.

Although metacognitive outcomes were not directly measured, the observed compression of lower-tail performance and stabilization of previously weak domains are consistent with instructional designs that promote self-regulated learning and targeted remediation. Importantly, the theoretical contribution of this work lies not in testing these theories per se, but in operationalizing them within a concrete, reproducible AI-enabled workflow.

### 4.5. ADAPT as an Enabling Infrastructure for Predictive Analytics

A central implication of this work is that ADAPT establishes the structural prerequisites for predictive analytics without prematurely advancing algorithmic claims. Diagnostic tiering, mastery mapping, and longitudinal alignment of assessment domains create the necessary data substrate for future forecasting models, while preserving interpretability and instructional relevance. Although implemented in a PharmD immunology course, ADAPT relies on diagnostic assessment, mastery mapping, and structured AI-guided remediation using standard LMS and assessment platforms, making the framework readily transferable to other health professions curricula. This framework may also be applied to interprofessional education in complex teams when learners need to constantly review specific portions of content based on complex memory retrieval and cognitive flexibility, that is, recall followed by repetition in a new learning context [20].

Rather than positioning prediction as an end in itself, this study demonstrates how diagnostic insight can be translated into immediate pedagogical action, a critical limitation of many learning analytics systems that identify risk without offering instructional response. In this sense, ADAPT functions as a bridge between formative assessment and predictive modeling, enabling future work to layer forecasting algorithms onto an already operational instructional framework.

### 4.6. Equity, Accessibility, and Linguistic Flexibility

An additional methodological contribution of ADAPT is its intentional support for multilingual AI interactions, allowing students to engage with tutoring prompts in a language of their choice. While language usage was not quantitatively analyzed, this design feature reflects a deliberate effort to reduce extraneous cognitive load and support a diverse learner population, including first-generation and international students.

This aspect of AI-enabled personalization remains underexplored in the literature and represents a promising avenue for future investigation, particularly in programs with linguistically diverse cohorts.

## 5. Limitations

Several limitations are inherent to the methodological scope of this work. The study was conducted within a single course at one institution, without randomization or control conditions. These limitations are consistent with the paper’s intent as a methods demonstration, rather than an outcomes trial. As an evaluation, it is a before-and-after study, and therefore, it is not appropriate to assume causality, as it is indeterminate what would have happened if AI had not been introduced. It is possible that other external factors, not captured within this evaluation, may explain the differences seen. Like all LLM-based tools, AI tutors carry risks of probabilistic errors and output variability; therefore, ADAPT was deliberately implemented as a faculty-supervised, assessment-anchored support system, with instructional authority and evaluative responsibility remaining solely with the instructor.

## 6. Conclusions

This study introduces ADAPT, an assessment-driven AI tutoring framework that enables personalized, adaptive learning using existing curricular infrastructure. By documenting the design logic, instructional sequencing, and empirical operation of the system, this work contributes a replicable blueprint for faculties seeking to integrate AI into health professions education without reliance on proprietary platforms or engineering teams. This is a novel idea, which is worthy of detailed investigation across several sites using experimental methods to enable causality to be confirmed.

## Data Availability

The original contributions presented in this study are included in the article (including Python codes created for this work). Further inquiries can be directed to the corresponding author.
